# Inhibition of MicroRNA-182/183 Cluster Ameliorates Schizophrenia by Activating the Axon Guidance Pathway and Upregulating DCC

**DOI:** 10.1155/2022/9411276

**Published:** 2022-11-10

**Authors:** Zhichao Wang, Lin Su, Tong Wu, Lei Sun, Zhenghai Sun, Yuchen Wang, Ping Li, Guangcheng Cui

**Affiliations:** ^1^Department of Academic Research, Qiqihar Medical University, Qiqihar 161000, China; ^2^Jiangxi Provincial Key Laboratory of Preventive Medicine School of Public Health, Nanchang University, Nanchang 330006, China; ^3^Department of Psychology, Qiqihar Medical University, Qiqihar 161000, China

## Abstract

Schizophrenia (SZ) is a complex disorder caused by a variety of genetic and environmental factors. Mounting evidence suggests the involvement of microRNAs (miRNAs) in the pathology of SZ. Accordingly, the current study set out to investigate the possible implication of the miR-182/183 cluster, as well as its associated mechanism in the progression of SZ. Firstly, rat models of SZ were established by intraperitoneal injection of MK-801. Moreover, rat primary hippocampal neurons were exposed to MK-801 to simulate injury of hippocampal neurons. The expression of miR-182/183 or its putative target gene DCC was manipulated to examine their effects on SZ *in vitro* and *in vivo*. It was found that miR-182 and miR-183 were both highly expressed in peripheral blood of SZ patients and hippocampal tissues of SZ rats. In addition, the miR-182/183 cluster could target DDC and downregulate the expression of DDC. On the other hand, inhibition of the miR-182/183 cluster ameliorated SZ, as evidenced by elevated serum levels of NGF and BDNF, along with reductions in spontaneous activity, serum GFAP levels, and hippocampal neuronal apoptosis. Additionally, DCC was found to activate the axon guiding pathway and influence synaptic activity in hippocampal neurons. Collectively, our findings highlighted that inhibition of the miR-182/183 cluster could potentially attenuate SZ through DCC-dependent activation of the axon guidance pathway. Furthermore, inhibition of the miR-182/183 cluster may represent a potential target for the SZ treatment.

## 1. Introduction

Schizophrenia (SZ) is a chronic mental disorder featured by signs of paranoid delusions and auditory hallucinations late in adolescence or early adulthood, affecting approximately 1% of the population across the world [1]. Fortunately, the hard-done work of our peers has highlighted changes in synaptic architecture and activity, gene expressions, and immunity, to serve as the major cellular mechanisms underlying the development of SZ [2]. Nevertheless, treatment of SZ remains a clinical challenge owing to its unknown etiology [3]. Therefore, it is imperative to expand our understanding of the molecular mechanisms underlying SZ in order to identify novel detection biomarkers and improve current treatment strategies.

MicroRNAs (miRNAs or miRs) are a group of short, noncoding RNAs that possess the ability to participate in the initiation and progression of neuropsychiatric disorders [4]. Interestingly, a number of miRNAs are known to exhibit abnormal expressions and dysfunction in the pathophysiology of SZ and are also highlighted as potential therapeutic targets for SZ [5]. Two such miRNAs, namely, miR-182 and miR-183, were previously found to be highly expressed in the course of SZ [6]. Moreover, the study performed by Rizos et al. indicated that upregulation of miR-183 may be associated with the initiation of SZ [7]. It is also noteworthy that there is evidence to suggest that miR-182 and miR-183 exert synergistic effects on a number of diseases [8, 9]. At this conjecture, we adopted a bioinformatics website and dual-luciferase reporter assay, which predicted and verified that the deleted in colon cancer (DCC) gene served as a target gene of the miR-182/183 cluster. Meanwhile, a prior study by Glasgow et al. has suggested that DCC loss in the pyramidal neurons at the hippocampal CA1 region led to impairments in spontaneous excitatory postsynaptic activity and ultimately damaged spatial memory [10]. DCC is associated with neuron development, synapse organization, and axon pathways [11]. On the other hand, low expressions of DCC were previously shown to promote SZ-like abnormalities in behaviors [11]. Besides, DCC is known to be significantly enriched in the axon guidance pathway, wherein the rs9944880 single nucleotide polymorphism (SNP) in DCC is associated with the pathogenesis of SZ [12]. Furthermore, DCC possesses three highly conserved protein-binding domains that mediate the assembly of multiple signaling components, which are essential for axon guidance cues [13]. More importantly, existing evidence indicates that genes implicated in axon guidance and synaptic transmission are more likely to contribute to SZ [14]. Accordingly, the current study set out to determine whether the miR-182/183 cluster would alleviate SZ through the axon guidance pathway by targeting DCC.

## 2. Materials and Methods

### 2.1. Ethics Statement

All clinical experiments were approved by the Clinical Ethics Committee of the Qiqihar Medical University and carried out in accordance with the *Declaration of Helsinki*. Signed informed consent was obtained from all participants and their caregivers prior to specimen collection. All animal protocols were approved by the Animal Ethics Committee of Qiqihar Medical University. Extensive efforts were made to minimize both the number and suffering of the experimental animals.

### 2.2. Study Subjects

The present study enrolled 59 patients with SZ and 48 normal controls. There were no significant statistical differences in sex and age between the SZ patients and normal controls (Supplementary Table 1). The 59 patients (32 males and 27 females; aged 10-65 years, with a mean age of 29.90 ± 12.49 years) with SZ were confirmed at the psychiatric outpatient department of the Qiqihar Fuyu Neuropsychiatric Hospital from December 2016 to December 2017. Inclusion criteria for the patients were as follows: (1) patients met the diagnostic criteria for SZ in the American Diagnostic and Statistical Manual of Mental Disorders, Fourth Edition (DSM-IV), and (2) patients were diagnosed with SZ for the first time or did not take antipsychotic medication 3 months prior to enrollment in the study. Meanwhile, the exclusion criteria for the patients were as follows: (1) patients suffered from other mental disorders, (2) patients suffered from physical or neurological disorders such as brain trauma, (3) patients with a history of drug or alcohol abuse, (4) patients with a history of blood transfusion 1 month prior to enrollment in the study, and (5) patients underwent treatment with modified electroconvulsive therapy (MECT) 3 months prior to enrollment in the study. Additionally, another 48 healthy individuals (25 males and 23 females, with a mean age of 33.90 ± 8.83 years), who took health examination in the Third Affiliated Hospital of Qiqihar Medical University were included as the normal controls. Inclusion criteria for the control subjects were as follows: (1) no family history of SZ, (2) no major traumatic events in the past month, or (3) no history of blood transfusion in the past month. Peripheral blood samples were collected from the patients and normal controls and stored at -80°C for further studies.

### 2.3. Establishment of SZ Rat Models

Seventy specific pathogen-free (SPF) adult male Sprague-Dawley (SD) rats (weighing 200-250 g, Liaoning Changsheng Biotechnology Co., Ltd., Benxi, China) were included in the current study. The procured rats were housed for at least 3 days before experiments in an animal facility at 22-24°C and 40-55% humidity under a 12 h light/dark cycle, with *ad libitum* access to food and water. After the initial acclimatization period, the rats were randomly classified into the SZ group (*n* = 60, rats were intraperitoneally injected with MK-801 at a dose of 5 mg/kg/day, once a day for 6 days) [15] and the normal group (*n* = 10, rats were administered with an equal volume of normal saline in the same way).

### 2.4. Stereotactic Injection

Rats were anesthetized by intraperitoneal injection of 10% chloral hydrate solution, calculating the injection volume of chloral hydrate as 30 mg/kg. Under deep anesthesia, the rats were fixed on the stereotaxic apparatus.. After disinfection with iovolt and 75% alcohol, the scalp was cut along the midline between the ears. The lateral skull was drilled stereographically at the antero-posterior 3.96 mm, mediolateral ±3.0 mm, and dorsoventral 3.0 mm, relative to the Bregma. Using a microinjection system (World Precision Instruments), AgomiR control, AgomiR-182/183, scrambled control, AntagomiR-182-183 (0.4 nmol dissolved in 2 *μ*L PBS), and AAV-DCC (2 *μ*L, 1 × 10^12^ TU/mL) were injected into the hippocampal CA1 region at a rate of 0.125 *μ*L/min. The needle was kept for 10 min before withdrawal, and the skin was sutured.

### 2.5. Animal Treatments

**Figure 1:**
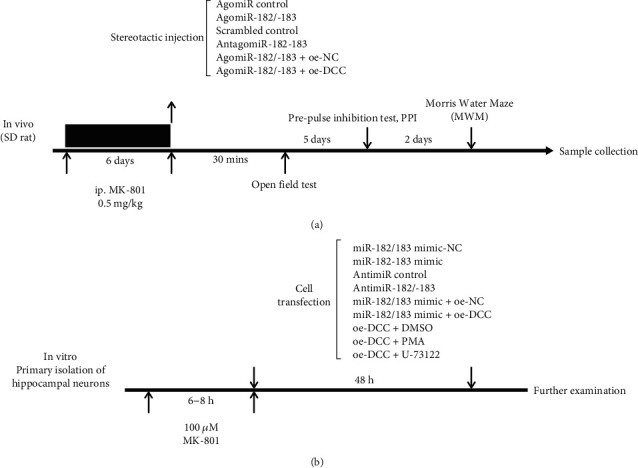
A schematic figure of the in vivo (a) and in vitro (b) experiment design.

Fifty-six rats that had been successfully modeled were randomly divided into the following 7 groups (*n* = 8/group): the SZ group (stereotactic injection of equal volume of normal saline after SZ modeling), the AgomiR-182/183 control group (stereotactic injection of AgomiR negative control (NC) into the hippocampus of rats), the AgomiR-182/183 group (stereotactic injection of miR-182/183 AgomiR into the hippocampus of rats), the scrambled control group (stereotactic injection of scrambled control into the hippocampus of rats), the AntagomiR-182/183 group (stereotactic injection of miR-182/183 AntagomiR into hippocampus), the AgomiR-182/183+oe-NC group (stereotactic injection of miR-182/183 AgomiR and DCC overexpression NC into hippocampus), and the AgomiR-182/183+oe-DCC groups (stereotactic injection of miR-182/183 AgomiR and adenoviral DCC overexpression into hippocampus). The transfection preparations were provided by GenePharma Co., Ltd. (Shanghai, China). After behavioral tests, rats were euthanized after orbital blood (2 mL) extraction. The rats were anesthetized, and the hippocampal tissues were harvested after behavioral tests. A schematic figure of the in vivo experiment design is shown in Figure 1(a).

### 2.6. Open Field Test

We used the open field test monitoring system (Xinruan Information Technology Co., Ltd, Shanghai, China) to assess spontaneous activity in rats. The rat cage was placed indoors before the experiment for 1 h to allow rats to adapt to the indoor environment. Rats were placed in an acrylic box (50 cm × 50 cm × 50 cm) and monitored for 10 min. The movement of the rats was detected by infrared sensors and recorded by a computer.

### 2.7. Prepulse Inhibition (PPI) Test

A background white noise of 70 dB was set and continued throughout the test. Rats were allowed to adapt to the environment for 5 min prior to testing. The PPI test was then performed every 15-25 s. The PPI test consisted of individual pulses (120 dB), prepulses (75 dB, 80 dB, and 85 dB), and a no-stimulus test (70 dB). The value of PPI was calculated as the percentage inhibition of shock amplitude caused by individual pulse: %PPI = 100% (amplitude of individual pulse − amplitude of (pre − pulse + pulse test)/amplitude of individual pulse).

### 2.8. Morris Water Maze (MWM) Test

In the 2^nd^ week of model construction, the learning ability and memory impairment of rats in each group were evaluated by MWM in the SZ rats based on existing protocols [16].

### 2.9. Nissl Staining

After behavioral tests, rats were euthanized, and brain tissues were isolated and sliced into 40 *μ*m thick coronal sections. Sections of the entire hippocampus were fixed on gelatin-coated slides and air-dried overnight. Sections were defibrobated in 0.01 m sodium citrate buffer (pH 6.0) and rehydrated, then microwaved for 5 min and cooled to room temperature. Sections were stained with cresol purple and dehydrated in 95% ethanol for 5 min, 100% ethanol for 10 min, and xylene for 10 min. Histological analysis was performed by three laboratory technicians blinded to the experimental groups.

### 2.10. Isolation and Culture of Primary Hippocampal Neurons

Rats at the 15^th^ day of gestation were anesthetized with an intraperitoneal injection of 10% chloral hydrate at a dose of 30mg/kg and then fixed on an experimental table. Under sterile conditions, the uterus was exposed and separated, and the fetuses were harvested. Fetuses were placed in a culture dish containing precooled sterile D-Hank's solution. The hippocampal tissues from these fetuses were carefully harvested under a dissecting microscope. Hippocampal tissues were then sliced into small sections and transferred to a centrifuge tube. Next, ten times volume of 0.125% trypsin was used to detach the tissues for 15 min in a 37°C oscillating water bath. The tissues were gently triturated several times to prepare a homogenous mixture with no visible clusters of cells. The reaction was terminated with the addition of an equivalent volume of Dulbecco's modified Eagle's medium (DMEM)/F12 containing 10% fetal bovine serum (FBS). After mixing, the cells were centrifuged at 1000 rpm for 5 min at room temperature, resuspended in DMEM/F12 medium containing 10% FBS, counted, and seeded at a density of 2 × 10^5^/cm^2^ in a culture plate precoated with 0.1 mg/mL of poly-l-lysine (PLL). Next, the cells were cultured at 37°C with 5% CO_2_ under 95% saturated humidity. The culture medium was replaced every 3 days. On the 2^nd^ day of culture, cytarabine (final concentration: 4 *μ*g/mL) was added to the cells to inhibit glial cell growth. Medium containing cytarabine was replaced after 24 h. The cells were continuously cultured for 5 days and used for subsequent experiments. A schematic figure of the *in vitro* experiment design is shown in Figure 1(b).

### 2.11. Cell Grouping and Transfection

The neurons were treated with either MK-801 (Sigma-Aldrich Chemical Co., St. Louis, MO) or serum/nutrient deprivation. MK-801 (100 *μ*M, dissolved in dimethyl sulfoxide (DMSO), Wako Pure Chemical Industries Ltd., Osaka, Japan) was added to the medium. The final concentration of DMSO was below 0.4%. Following exposure to 100 *μ*M MK-801, the neurons were assigned into the following groups: the miR-182/183 mimic-NC group (MK-801-exposed neurons were transfected with miR-182/183 cluster overexpression NC sequence), the miR-182/183 mimic group (MK-801-exposed neurons were transfected with miR-182/183 cluster overexpression sequence), the AntimiR-182/183 control group (MK-801-exposed neurons were transfected with miR-182/183 cluster interference NC sequence), the AntimiR-182/183 group (MK-801-exposed neurons were transfected with miR-182/183 cluster interference sequence), the oe-NC group (MK-801-exposed neurons were transfected with DCC overexpression NC sequence), the oe-DCC group (MK-801-exposed neurons were transfected with DCC overexpression sequence), the miR-182/183 mimic+oe-NC group (MK-801-exposed neurons were transfected with miR-182/183 cluster overexpression sequence and DCC overexpression NC sequence), the miR-182/183 mimic+oe-DCC group (MK-801-exposed neurons were transfected with miR-182/183 cluster and DCC overexpression sequence), the oe-DCC+DMSO group (MK-801-exposed neurons were transfected with DCC overexpression sequence and phosphoinositide-specific phospholipase C (PLC) pathway control solvent), the oe-DCC+PMA group (MK-801-exposed neurons were transfected with DCC overexpression sequence and PLC pathway activator, PMA), and the oe-DCC+U-73122 group (MK-801-exposed neurons were transfected with DCC overexpression sequence and PLC pathway inhibitor, U-73122). Subsequently, the cells were incubated at 37°C with 5% CO_2_ for 6-8 h. The original culture medium was replaced with complete culture medium where the cells were further cultured for 48 h and collected for subsequent experiments.

### 2.12. Bioinformatics Analysis

The RNA22 database was adopted to precisely predict the downstream target genes of miR-182 and miR-183, followed by the intersection of the prediction results. The “org.Hs.eg.db” installation package of R language was utilized to convert the obtained gene symbol to entrezIDs. Subsequently, functional enrichment analysis of the potential target genes of miR-182/183 was performed using the “clusterprofiler” package.

### 2.13. Dual-Luciferase Reporter Assay

Human embryonic kidney HEK293T cells were cultured in DMEM containing 10% FBS at 37°C with 5% CO_2_. Subsequently, the 3′-untranslated region (3′-UTR) fragment in DCC containing the miR-182/183 binding site was inserted into the pmirGLO vector. The mutant DCC 3′-UTR fragment of the binding site was constructed by site-directed mutagenesis and inserted into the pmirGLO vector. The recombinant plasmid pmirGLO-DCC or pmirGLO-mut DCC was then cotransfected into the HEK293T cells with miR-182/183 mimic or miR-NC using the liposome transfection method. The cells were then cultured for 48 h, collected, and lysed. The lysate supernatant (100 *μ*L) was mixed with an equal volume of Renilla luciferase assay solution. Another 100 *μ*L of lysate supernatant was mixed with an equal volume of the firefly luciferase assay reagent. The Renilla and firefly luciferase activities were detected within 10 s at an interval of 2 s using the SpectraMax M5 multifunction microplate reader.

### 2.14. Hoechst 33342 Staining

Cell apoptosis was measured using Hoechst 33342/propidium iodide (PI) double staining kit (CA1120, Solarbio Science & Technology Co., Ltd., Beijing, China). Briefly, the cells (1 × 10^6^) in each sample were centrifuged, and the supernatant was discarded. The cell pellets were resuspended in 1 mL cell staining buffer. Next, the cells were added with 5 *μ*L of Hoechst staining solution and 5 *μ*L of PI staining solution, mixed, and incubated at 4°C for 20-30 min. Red and blue fluorescence channels were detected using flow cytometry under a fluorescence microscope.

### 2.15. RNA Isolation and Quantification

Total RNA in the cells and tissues was extracted using the TRIzol reagent (Takara Biotechnology Ltd., Dalian, China), and the concentration and purity were determined. The obtained total RNA was reverse-transcribed into cDNA using the PolyA Tailing Reverse Transcription Kit (B532451, Sangon Biotech Co., Ltd., Shanghai, China). Primer sequences of miR-182/183 and DCC are shown in Supplementary Table 2 (Shanghai Genechem Co., Ltd., Shanghai, China). mRNA expression was measured using a fluorescence quantitative PCR kit (Takara Biotechnology). U6 was employed as an internal reference for miR-182/183, while glyceraldehyde-3-phosphate dehydrogenase (GAPDH) was adopted for the remaining genes. Relative expression of target genes was calculated using the 2^-*ΔΔ*Ct^ method.

### 2.16. Western Blot Analysis

Total protein content was extracted from cells or tissues, with the concentration determined with a bicinchoninic acid (BCA) protein assay kit (23225, Pierce, Rockford, IL). The obtained protein was then separated by 10% sodium dodecyl sulfate-polyacrylamide electrophoresis (SDS-PAGE, P1200, Solarbio) and transferred onto a polyvinylidene fluoride (PVDF) membrane using the semidry electrophoretic transfer method. After two washes with Tris-buffered saline Tween 20 (TBST), the membrane was blocked with 5% skim milk at room temperature for 2 h and then incubated with the following primary antibodies (Abcam Inc., Cambridge, UK): rabbit polyclonal antibody PLC*γ* (dilution ratio of 1 : 1000, ab109501), rabbit antibody DCC (dilution ratio of 1 : 1000, ab273570), and rabbit antibody GAPDH (dilution ratio of 1 : 500, ab9485) at 4°C overnight. After another three washes with TBST (10 min/time), the membrane was incubated with the horseradish peroxidase- (HRP-) labeled secondary antibody goat anti-rabbit immunoglobulin G (IgG) (dilution ratio of 1 : 2000, ab6721, Abcam) at room temperature for 2 h. Subsequently, the membrane was developed with DAB and photographed with a gel imager (Gel Doc XR, Bio-Rad, Hercules, CA). Relative expression of the target protein was calculated as the ratio between the gray value of the target protein and internal reference GAPDH. The aforementioned method was equally applicable to protein expression detection in cells.

### 2.17. Enzyme-Linked Immunosorbent Assay (ELISA)

Serum samples from the SZ patients and rats were collected to determine circulating levels of nerve growth factor (NGF; tw041535, Tongwei Industrial Co., Ltd., Shanghai, China), brain-derived neurotrophic factor (BDNF; tw040860, Tongwei Industrial Co., Ltd.), and glial fibrillary acidic protein (GFAP; ml262817, Shanghai Enzyme-linked Biotechnology Co., Ltd., Shanghai, China) by ELISA kits according to the instructions.

The cell supernatant was collected to determine the concentration of phosphatidylinositol 4,5-bisphosphate (PIP_2_; JLC009-96T, Jingkang Biological Engineering Co., Ltd., Shanghai, China) and inositol 1,4,5-trisphosphate (IP_3_; JL50125-96T, Jianglai Biological Technology Co., Ltd., Shanghai, China) in strict accordance with the provided instructions of the ELISA kits. Optical density (OD) was measured at an excitation wavelength of 450 nm using a microplate reader (BS-1101, Detie Experimental Equipment Co., Ltd., Nanjing, China).

### 2.18. Statistical Analysis

Statistical analysis was performed using the SPSS 21.0 software (IBM Corp., Armonk, NY). Measurement data were expressed as the mean ± standard deviation. Data with normal distribution and homogeneity of variance between two groups were compared using the independent sample *t*-test. Comparison among multiple groups was conducted by one-way analysis of variance (ANOVA), followed by Tukey's post hoc test. A value of *p* < 0.05 was regarded as statistically significant.

## 3. Results

### 3.1. miR-182 and miR-183 Highly Expressed in SZ

**Figure 2:**
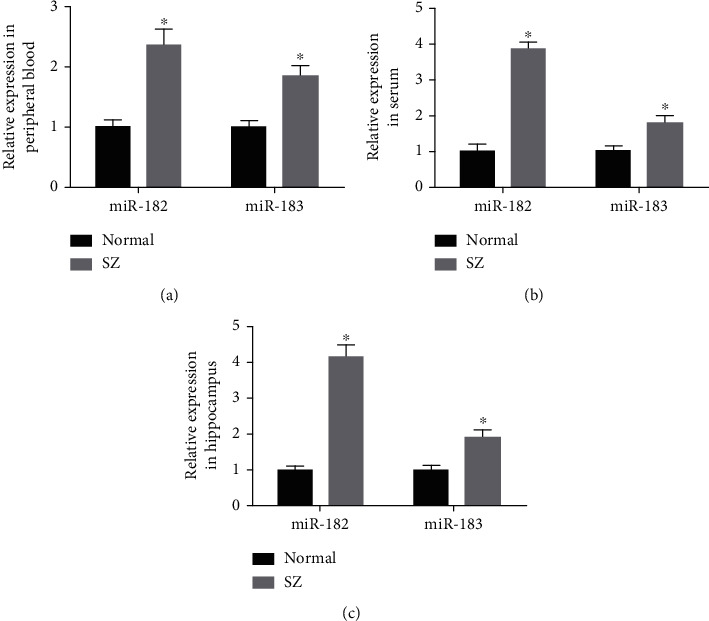
miR-182 and miR-183 are highly expressed in SZ. (a) RT-qPCR was carried out to measure the expression of miR-182/183 in the peripheral blood sample of SZ patients (*n* = 59) and healthy individuals (*n* = 48). (b) RT-qPCR was conducted to determine the expression of miR-182/183 in the serum samples from SZ (*n* = 8) and normal (*n* = 8) rats. (c) RT-qPCR was performed to determine the expression of miR-182/183 in the hippocampal tissues from SZ (*n* = 8) and normal (*n* = 8) rats. The experimental data were measurement data, expressed as the mean ± standard deviation, and analyzed by independent sample *t*-test. ^∗^*p* < 0.05*vs*. the normal group.

Firstly, we uncovered a total of 322 differentially expressed miRNAs following miRNA expression microarray sequencing in SZ patients and healthy individuals [6]. Interestingly, both miR-182 and miR-183 were found to be among the top 50 most differentially expressed miRNAs. Meanwhile, existing studies have shown that miR-182 and miR-183 belong to the same miRNA cluster and have synergistic functions [8, 9, 17, 18]. In addition, the results of RT-qPCR results illustrated higher expression of miR-182 and miR-183 in the peripheral blood samples from SZ patients than that from healthy individuals (Figures 1(a) and 2(a)). The SZ rat models were successfully established (Supplementary Figure 1). Moreover, miR-182 and miR-183 expression was also elevated in the serum and hippocampal tissues of SZ rats (Figures 2(b) and 2(c)). Together, these findings suggested that miR-182 and miR-183 were highly expressed in SZ.

Analysis of the relationship between different clinicopathological characteristics of SZ patients and miR-182 and miR-183 expression revealed no significant difference between miR-182 and miR-183 expression and the gender and ages of SZ patients, whereas a significant difference was noted between the clinical stages, predisposing factors, and genetic family history and miR-182 and miR-183 expressions (Supplementary Table 1). Altogether, the aforementioned findings indicated that both miR-182 and miR-183 were abundantly expressed in SZ, and their expression may be correlated with the patient's clinical classification, predisposing factors, and genetic family history.

### 3.2. Inhibition of miR-182/183 Cluster Retards Development and Progression of SZ

**Figure 3:**
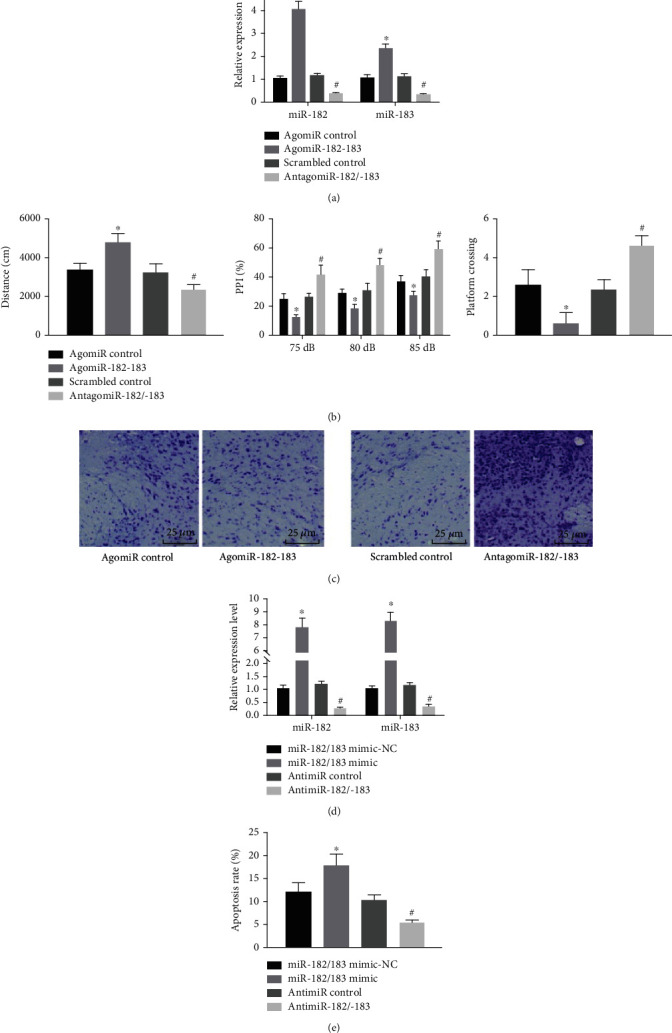
Inhibition of miR-182/183 cluster improves SZ at different levels: (a) miR-182/183 cluster transfection efficiency (*n* = 8/group); (b) open field test (left), PPI test (middle), and Morris water maze test (right) in SZ rats (*n* = 8/group); (c) representative micrographs showing Nissl body in hippocampal tissues from SZ rats. MK-801-exposed hippocampal neurons were treated with miR-182/183 mimic or AntimiR-182/183; (d) expression of miR-182 and miR-183 in the MK-801-exposed hippocampal neurons determined by RT-qPCR; (e) quantitative analysis of apoptosis of MK-801-exposed hippocampal neurons detected by Hoechst 33342 staining. The data in the figure were measurement data, expressed as the mean ± standard deviation, and analyzed by one-way ANOVA. ^∗^*p* < 0.05*vs.* the AgomiR control or miR-182/183 mimic-NC control; ^#^*p* < 0.05*vs*. the scrambled control or AgomiR control group. The cell experiments were repeated three times independently.

To further elucidate the effects of the miR-182/183 cluster on the biological functions of SZ rats, the rats were injected with AgomiR- and AntagomiR-182/183 (Figure 3(a), Supplementary Figure 2). Subsequent observation of the behavioral changes of rats in each group revealed that the total distance of spontaneous movement (left panel, Figure 3(b)) was reduced, while PPI for all prepulse intensities (75 dB, 80 dB, and 85 dB) (middle panel, Figure 3(b)) and the number of crossing the underwater platform (right panel, Figure 3(b)) were both increased in the AntagomiR-182/183 group. Moreover, ELISA was adopted to measure the serum levels of NGF, BDNF, and GFAP of rats in each group. The results showed that the serum levels of NGF and BDNF were increased, while GFAP levels were decreased in the AntagomiR-182/183 compared to those in the scrambled control group (Supplementary Table 3).

Furthermore, we observed the number of Nissl's body in the hippocampal tissues with Nissl staining. It was found that Nissl body exhibited dark-blue plaque with a large amount of distribution (Figure 3(c)).

Next, the effect of the miR-182/183 cluster on the injury of hippocampal neurons induced by MK-801 was determined at the cellular level. The results of RT-qPCR demonstrated that miR-182 and miR-183 expression was increased in the MK-801-exposed neurons treated with miR-182/183 mimic, while an opposite result was noted in the presence of AntimiR-182/183 (Figure 3(d)).

In addition, the results of Hoechst 33342 staining illustrated that apoptosis of hippocampal neurons was significantly lower following AntimiR-182/183 compared to its control (Figure 3(e), Supplementary Figure 3A). Collectively, the aforementioned findings indicated that inhibition of the miR-182/183 cluster may improve SZ at different levels.

### 3.3. DCC Is a Target Gene of miR-182/183 Cluster

**Figure 4:**
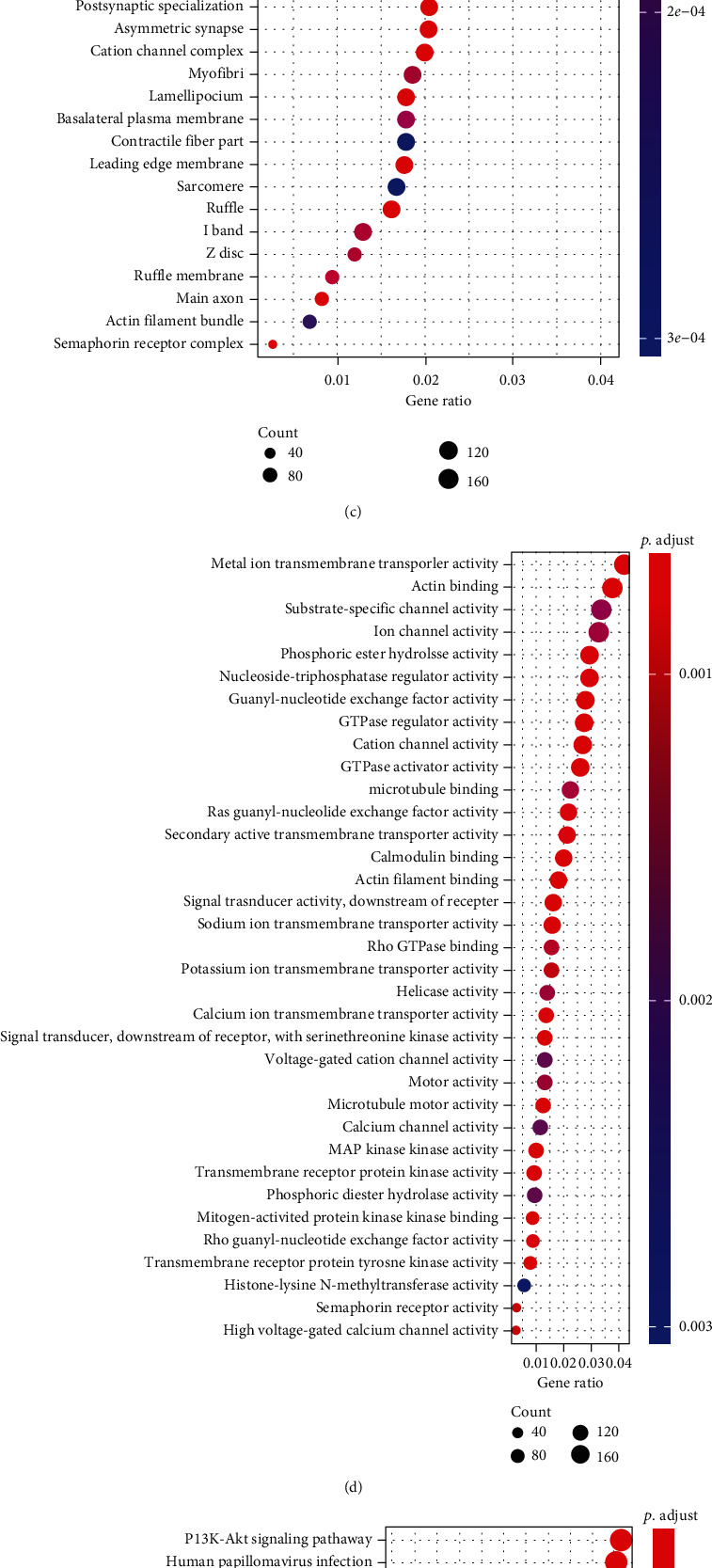
miR-182/183 targets DCC and negatively regulates DCC: (a) Venn diagram of the predicted target genes of miR-182 and miR-183 by the RNA22 database. The blue circle represents the prediction result of miR-182, the red circle represents the prediction result of miR-183, and the middle part represents the intersection of the two sets of data. (b–d) GO function analysis of miRNA target gene prediction results; (b–d) enrichment results of three GO functional groups: biological process (BP), cellular component (CC), and molecular function (MF). The abscissa indicates the generation, and the ordinate indicates the name of entry identifiers. The circle size and color in the figure indicate the number of genes in the entry identifier and the *p* value, respectively. (e) KEGG enrichment analysis of metabolic pathway in relation to miRNA target gene prediction results; (f) Western blot analysis of DCC protein in the hippocampal tissues of SZ (*n* = 8) and normal (*n* = 8) rats; (g) the binding site between miR-182/183 and DCC predicted by the bioinformatics website https://cm.jefferson.edu/rna22/; (h) binding of miR-182/183 to DCC determined by dual-luciferase reporter assay; (i) Western blot analysis of DCC protein in the hippocampal neurons treated with miR-182/183 mimic or AntimiR-182/183. The data in the figure were measurement data, expressed as the mean ± standard deviation, and analyzed by an independent sample *t*-test. ^∗^*p* < 0.05*vs.* the normal or miR-182/183 mimic-NC group. ^#^*p* < 0.05*vs.* the AntimiR control group. The cell experiments were repeated three times independently.

To further explore the mechanism of the miR-182/183 cluster in SZ, the RNA22 database was first used to predict the downstream target genes of the two miRNAs, which were then intersected, with 4,641 potential regulatory target genes obtained (Figure 4(a)). Subsequent GO and KEGG enrichment analysis suggested that these genes were primarily enriched in “axon development,” “actin cytoskeleton,” “metal ion transmembrane transporter activity,” and the “PI3K-Akt signaling pathway” (Figures 4(b)–4(e)). Among them, axon development was selected as our focus due to the close association of the axon signaling pathway with SZ development [19, 20]. Moreover, we also found that these genes were mainly enriched in pathways such as axonal development. For instance, the DCC gene was mainly enriched in “axonogenesis” and “axon guidance.” Besides, existing studies have highlighted the possible involvement of DCC in the regulation of SZ [11, 21]. Together, these findings suggested that the miR-182/183 cluster was highly likely to regulate SZ development by targeting DCC *via* the axon guidance pathway.

For verification, we first performed Western blot analysis to detect the protein expression patterns of DCC in the hippocampal tissue of rats in each group. The results illustrated significantly lower protein expression of DCC in the hippocampal tissue of SZ rats than that in the normal rats (Figure 4(f)). Additionally, the bioinformatics website (https://cm.jefferson.edu/rna22/) suggested the presence of potential binding sites in miR-182 (upper panel, Figure 4(g)) and miR-183 (lower panel, Figure 4(g)) for DCC. Furthermore, the results of the dual-luciferase reporter assay (Figure 4(h)) showed that compared with the miR-182/183 mimic-NC group, the luciferase activity of DCC wild-type 3′-UTR was reduced in the miR-182/183 mimic group, while that of mutant 3′-UTR was unaffected. Meanwhile, Western blot analysis results also illustrated a decline in the protein expression of DCC in the hippocampal neurons in the miR-182/183 mimic group relative to the miR-182/183 mimic-NC. In contrast, the protein expression of DCC was elevated in the AntimiR-182/183 group as compared to the AntimiR control group (Figure 4(i)). Altogether, these findings provided evidence that miR-182/183 targeted DCC and downregulated its expression.

### 3.4. miR-182/183 Cluster Enhances SZ by Downregulating DCC

**Figure 5:**
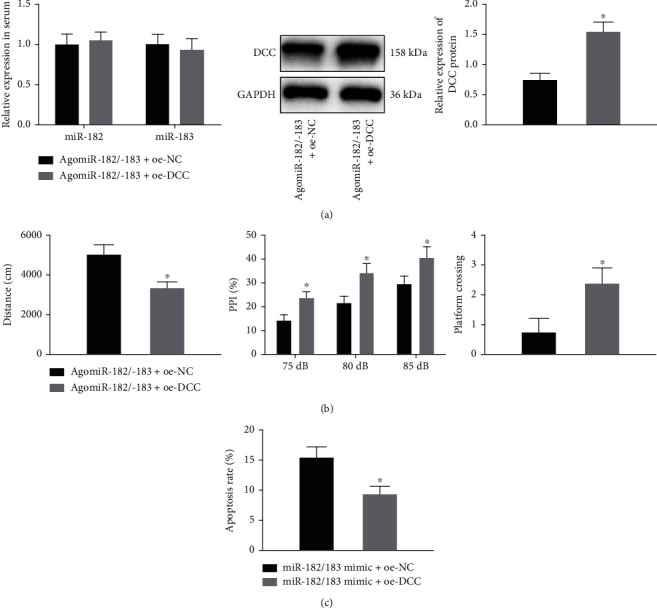
DCC overexpression reverses the promoting effect of the miR-182/183 cluster on the progression of SZ: (a) expression of miR-182 and miR-183 in the hippocampal tissues of SZ rats with AgomiR-182/183+oe-DCC determined by RT-qPCR along with Western blot analysis of DCC protein in the hippocampal tissues of SZ rats with AgomiR-182/183+oe-DCC (*n* = 8/group); (b) open field test (left), PPI test (middle), and Morris water maze test (right) in SZ rats (*n* = 8/group); (c) apoptosis in hippocampal neurons in response to AgomiR-182/183+oe-DCC. The data in the figure were measurement data, expressed as the mean ± standard deviation, and analyzed by an independent sample *t*-test. ^∗^*p* < 0.05*vs.* the AgomiR-182/183+oe-NC or miR-182/183 mimic+oe-NC group. The cell experiments were repeated three times independently.

In order to further understand the effect of the miR-182/183 cluster on SZ rats' biological functions *via* regulation of DCC, SZ rats were treated with AgomiR-182/183 or in combination with oe-DCC. RT-qPCR and Western blot analysis results presented no changes in miR-182 and miR-183 expression yet an elevation in that of DCC in the hippocampal tissue of SZ rats in the AgomiR-182/183+oe-DCC group compared with AgomiR-182/183 alone (Figure 5(a)). In addition, behavioral test results showed that compared with AgomiR-182/183 alone, the total distance of spontaneous movement was significantly reduced (left panel, Figure 5(b)), while PPI for all prepulse intensities (75 dB, 80 dB, and 85 dB) (middle panel, Figure 5(b)) and the number of platform crossings (right panel, Figure 5(b)) were remarkably increased in the AgomiR-182/183+oe-DCC group.

Furthermore, the results of Hoechst 33342 staining illustrated that apoptosis in hippocampal neurons in the miR-182/183 mimic+oe-DCC group was significantly lower than that in the miR-182/183 mimic+oe-NC group (Figure 5(c), Supplementary Figure 3B). Overall, the abovementioned findings indicated that DCC overexpression could reverse the promoting effect of the miR-182/183 cluster on the progression of SZ.

### 3.5. DCC Activates the Axon Guidance Pathway in Hippocampal Neurons

**Figure 6:**
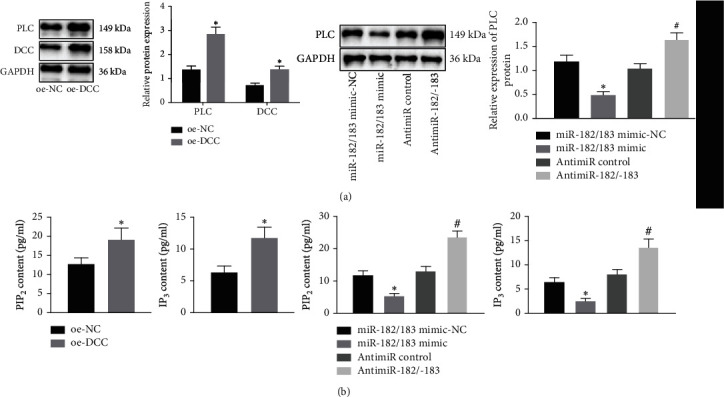
DCC activates the axon guidance pathway and thus affects synaptic activity in hippocampal neurons. (a) Expression of DCC and axon guidance pathway-related protein PLC in hippocampal neurons. (b) ELISA was used to detect the expression of PIP_2_ and IP_3_ in hippocampal neurons. The data in the figure were measurement data, expressed as the mean ± standard deviation. Comparison between two groups was analyzed by the independent sample *t*-test; comparison among multiple groups was analyzed by one-way ANOVA, followed by Tukey's post hoc test. ^∗^*p* < 0.05*vs*. the oe-NC, miR-182/183 mimic NC, or oe-DCC+DMSO group. ^#^*p* < 0.05*vs*. the AntimiR control group. The cell experiments were repeated three times independently.

Axon guidance is recognized as a special form of movement during the course of nerve cell development. Through its expanded structure at the end—the receptor on the surface of the growth cone, it possesses the ability to recognize the signal molecules expressed in different time and space on the growth path, isolate the target, and obtain the signal to stop its forward movement, so as to establish a synaptic connection between axons and target cells. Furthermore, existing evidence suggests that axon guidance pathway-related factors include PLC, PIP_2_, and IP_3_ [22]. In the above studies, we found that DCC was mainly enriched in pathways such as “axonogenesis” and “axon guidance.” Therefore, we speculated whether DCC mediates axon guidance and synapse specificity to affect neuronal signals, thereby regulating SZ. MK-801-exposed hippocampal neurons were treated with miR-182/183 mimic, AntimiR-182/183, or oe-DCC. The results of Western blot analysis exhibited that the expression of axon-guidance pathway-related protein PLC was significantly increased in hippocampal neurons from the oe-DCC group when compared to the oe-NC group. Meanwhile, in the miR-182/183 mimic group, PLC protein expression was found to be decreased relative to the miR-182/183 mimic NC group. In addition, PLC protein expression was elevated in the AntimiR-182/183 compared to the AntimiR control group (Figure 6(a)). Moreover, ELISA data revealed that the levels of PIP_2_ and IP_3_ were both increased in hippocampal neurons from the oe-DCC group when compared to those in the oe-NC group. In contrast, PIP_2_ and IP_3_ levels were found to be lower in the miR-182/183 mimic group than those in the miR-182/183 mimic NC group. Besides, the AntimiR-182/183 group presented with increased levels of PIP_2_ and IP_3_ in comparison to the AntimiR control group (Figure 6(b)). Overall, these findings indicated that DCC could activate the axon guidance pathway, while the miR-182/183 cluster negatively regulated the axon guidance pathway.

## 4. Discussion

SZ is a severe psychiatric disorder accompanied by a complex array of signs and symptoms, resulting in a significant impact on the quality of life of patients and huge economic burdens on society [23]. Thus, it is prudent to keep expanding the search for the underlying mechanisms of SZ in order to help identify novel therapeutic targets. Accordingly, the current study illuminated the role of the miR-182/183 cluster in the synaptic activity of SZ, and the obtained findings revealed that suppression of the miR-182/183 cluster could promote synaptic activity in hippocampal neurons in SZ through DCC-dependent activation of the axon guidance signaling pathway.

Initial findings of this study revealed that both miR-182 and miR-183 were highly expressed in SZ. As highly homologous miRNAs, miR-182 and miR-183 are known to function as a gene cluster (miR-182/183 cluster) and further exert important roles in human malignancies [17, 18]. Meanwhile, previous studies have highlighted the ability of miRNAs to serve as key regulators in the developmental processes in SZ [5]. Consistent with our findings, a prior study also came across upregulation of both miR-182 and miR-183 expression in SZ [6]. These findings and evidence were indicative of the close involvement of the miR-182/183 cluster in SZ progression. Further analysis of this study demonstrated that inhibition of the miR-182/183 cluster brought about an improvement in SZ, as evidenced by decreased serum GFAP levels and increased NGF and BDNF levels. Our findings are in agreement with a previous study illustrating that GFAP, a well-established prototypical astrocyte marker, was upregulated in the prefrontal cortex from SZ patients [24]. Moreover, there is also evidence to suggest that miR-182 upregulation is associated with decreased BDNF expression in the hippocampal tissues of chronic unpredictable mild stress models [25]. In addition, overexpression of the miR-182/183 cluster results in progressive neurosensory hearing loss [26].

Another important finding in our study revealed that miR-182/183 targeted DCC and downregulated the expression of DCC. In addition, our results demonstrated that DDC overexpression resulted in the activation of the axon guidance pathway. It is noteworthy that DCC is essential for CB1 receptor-mediated growth cone reorganization in the process of axon guidance and development [27]. Additionally, DCC serves as a receptor for the guidance cue netrin-1 and plays an essential role in organizing neuronal circuitry by guiding growing axons and dendrites to their correct targets and affecting synaptic connectivity [28]. A previous study has demonstrated that miR-182 can also play a role in the regulation of axon guidance [29]. Altogether, all the aforementioned findings are suggestive of DCC serving as a target gene of miR-182/183 in SZ.

Additionally, we further uncovered that miR-182/183 cluster inhibition exerted a promotive effect on synaptic activity in hippocampal neurons in SZ through DCC-mediated axon guidance pathway activation, as evidenced by reduced apoptosis, in hippocampal neurons. Similarly, a prior study indicated that changes in synaptic activity or entire tracts may lead to disturbances in mitochondrial density [30]. Meanwhile, IP3, an axon guidance pathway-related factor, was previously highlighted as a global messenger to enhance cytosolic Ca^2+^ concentration, such that the PLC-IP3 pathway is essential for the attractive turning of growth cones toward NGF [31]. Existing evidence further suggests that inhibition of miR-183/96/182 can attenuate the reprogramming of human retinal pigment epithelial cells to retinal neuron fate [32]. On the other hand, overexpression of miR-183 leads to the inhibition of hippocampal neural stem cell proliferation and differentiation in newborn rats [33]. Collectively, these findings and evidence demonstrated that inhibition of the miR-182/183 cluster could promote synaptic activity in hippocampal neurons in SZ *via* the activation of the axon guidance pathway by upregulating DCC.

**Figure 7:**
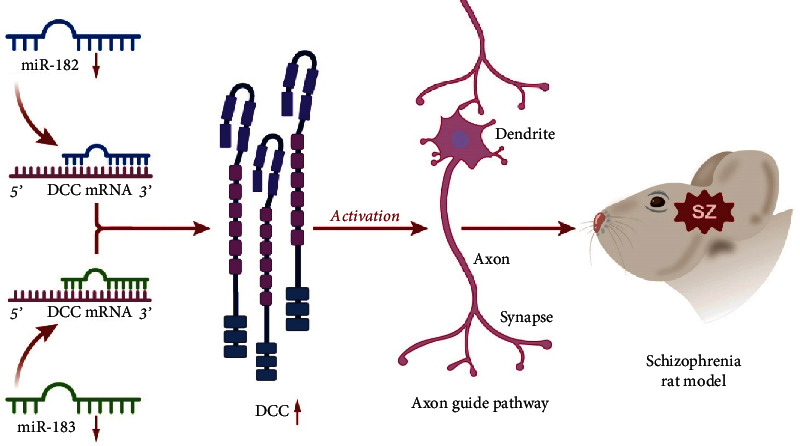
Schematic diagram of the mechanism by which miR-182/183 cluster affects SZ. miR-182 and miR-183 are highly expressed in SZ. Inhibition of miR-182/183 cluster can upregulate DCC, thereby activating the axon guidance pathway and ultimately preventing the occurrence and development of SZ.

In conclusion, the current study demonstrated that miR-182/183 was highly expressed in SZ, whereas suppression of the miR-182/183 cluster promoted synaptic activity in hippocampal neurons in SZ through the activation of the axon guidance pathway *via* upregulation of DCC (Figure 7). Therefore, these results provide new sights into the molecular mechanism involved in the development and progression of SZ. Targeting the miR-182/183 cluster may be a potential therapeutic target for SZ treatment.

## Data Availability

The data that support the findings of this study are available from the corresponding authors upon reasonable request.

## Supplementary Materials

Supplementary Materials Supplementary Figure 1: characterization of SZ rat models by open field test. Supplementary Figure 2: representative image of stereotactic injection. Supplementary Figure 3: representative micrographs. Supplementary Table 1: relationship between miR-182/183 expression and clinicopathological characteristics of SZ patients. Supplementary Table 2: primer sequences for RT-qPCR. Supplementary Table 3: serum levels of NGF, BDNF, and GFAP in different treatment groups of SZ rats.
