# Supplementary material for: Inhibition of MicroRNA-182/183 Cluster Ameliorates Schizophrenia by Activating the Axon Guidance Pathway and Upregulating DCC
Source: Oxid Med Cell Longev. 2022 Nov 10;2022:9411276. doi: 10.1155/2022/9411276 (PMC9671740; doi:10.1155/2022/9411276)
Supplement: Supplementary Materials — Supplementary Figure 1: characterization of SZ rat models by open field test. Supplementary Figure 2: representative image of stereotactic injection. Supplementary Figure 3: representative micrographs. Supplementary Table 1: relationship between miR-182/183 expression and clinicopathological characteristics of SZ patients. Supplementary Table 2: primer sequences for RT-qPCR. Supplementary Table 3: serum levels of NGF, BDNF, and GFAP in different treatment groups of SZ rats. [file 9411276.f1.zip › 9411276.f1.docx]

**Supplementary Table 1.** Relationship between miR-182/183 expression and clinicopathological characteristics of SZ patients

| Characteristics | Cases | miR-182 | *p* value | miR-183 | *p* value |
| --- | --- | --- | --- | --- | --- |
| Age (years) | 59 |  |  |  |  |
| ≤ 20 | 13 | 2.325 ± 0.302 | 0.706 | 1.900 ± 0.188 | 0.647 |
| 21 - 30 | 27 | 2.407 ± 0.255 |  | 1.846 ± 0.197 |  |
| 31 - 40 | 9 | 2.318 ± 0.355 |  | 1.799 ± 0.139 |  |
| 41 - 50 | 4 | 2.505 ± 0.069 |  | 1.909 ± 0.131 |  |
| > 50 | 6 | 2.365 ± 0.147 |  | 1.898 ± 0.093 |  |
| Gender |  |  |  |  |  |
| male | 32 | 2.332 ± 0.292 | 0.150 | 1.822 ± 0.173 | 0.067 |
| female | 27 | 2.432 ± 0.222 |  | 1.905 ± 0.167 |  |
| Clinical stages |  |  |  |  |  |
| introverted | 42 | 2.432 ± 0.261 | 0.038 | 1.900 ± 0.153 | 0.01 |
| extroverted | 9 | 2.211 ± 0.254 |  | 1.721 ± 0.200 |  |
| intermediated | 8 | 2.281 ± 0.209 |  | 1.804 ± 0.172 |  |
| Predisposing factor |  |  |  |  |  |
| yes | 34 | 2.451 ± 0.244 | 0.013 | 1.923 ± 0.182 | 0.024 |
| no | 25 | 2.279 ± 0.265 |  | 1.775 ± 0.119 |  |
| Family history of hereditary diseases |  |  |  |  |  |
| yes | 13 | 2.587 ± 0.123 | 0.001 | 1.984 ± 0.125 | 0.003 |
| no | 46 | 2.319 ± 0.265 |  | 1.825 ± 0.171 |  |

Notes: The experimental data were measurement data, expressed as mean ± standard deviation, and analyzed by independent samples *t* test or one-way ANOVA, followed by Tukey’s post-hoc test.

**Supplementary Table 2.** Primer sequences for RT-qPCR

| Gene | Sequences (5’ - 3’) |
| --- | --- |
| miR-182  (human) | Forward: 5′-GAGAACAGCAGGTCCAGCAT-3′ |
|  | Reverse: Universal reverse primer |
| miR-182 | Forward: 5′-TTTGGCAATGGTAGAACTCACACCG-3′ |
| (rat) | Reverse: Universal reverse primer |
| miR-183  (human) | Forward: 5′-GCGGCGGTATGGCACTGGTAGA-3′ |
|  | Reverse: Universal reverse primer |
| miR-183 | Forward: 5′-TATGGCACTGGTAGAATTCAC-3′ |
| (rat) | Reverse: Universal reverse primer |
| U6  (human) | Forward: 5′-GGGCAGGAAGAGGGCCTAT-3′ |
|  | Reverse: Universal reverse primer |
| U6  (rat) | Forward: 5′-CGTTATGTAGGCACCGCCTT-3′ |
|  | Reverse: Universal reverse primer |

Note: RT-qPCR, reverse transcription quantitative polymerase chain reaction; miR-182, microRNA-182; miR-183, microRNA-183; DCC, deleted in colorectal cancer; GAPDH, glyceraldehyde-3-phosphate dehydrogenase.

**Supplementary Table 3.** Serum levels of NGF, BDNF, and GFAP in different treatment groups of SZ rats

| Groups | AgomiR-182/183 control | AgomiR-182/183 | scrambled control | AntagomiR-182/183 |
| --- | --- | --- | --- | --- |
|  |  |  |  |  |
| NGF | 33.47 ± 4.75 | 25.11 ± 2.27^*^ | 31.62 ± 2.80 | 46.45 ± 3.78^#^ |
| BDNF | 182.69 ± 9.37 | 94.69 ± 7.16^*^ | 177.33 ± 10.84 | 245.68 ± 12.14^#^ |
| GFAP | 289.83 ± 20.68 | 390.66 ± 28.41^*^ | 267.01 ± 28.59 | 171.32 ± 15.97^#^ |

Notes: The data are measurement data, expressed as mean ± standard deviation, and analyzed by one-way ANOVA followed by Tukey’s post-hoc test. ^*^*p* < 0.05 *vs.* AgomiR-182/183 control, ^#^ *p* < 0.05 *vs*. the scrambled control group. n = 8 for rats in each group.

**SUPPLEMENTARY FIGURE 1:** Characterization of SZ rat models by open field test (A), PPI test (B), and Morris mater maze test (C).


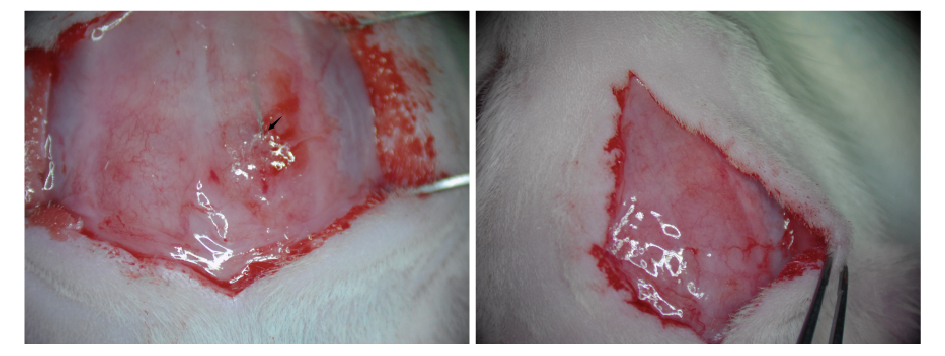


**SUPPLEMENTARY FIGURE 2:** Representative image of stereotactic injection.

**SUPPLEMENTARY FIGURE 3:** Representative micrographs. A representative micrographs showing apoptosis of MK-801-exposed hippocampal neurons detected by Hoechst 33342 staining. B, representative micrographs showing MTP in the MK-801-exposed hippocampal neurons determined by JC-1 staining. C, representative micrographs showing changes in mitochondrial Ca^2+^ determined by Ca^2+^ indicator Rhod-2 AM. D, representative micrographs showing apoptosis in hippocampal neurons in response to AgomiR-182/183 + oe-DCC. E, representative micrographs showing MTP in hippocampal neurons in response to AgomiR-182/183 + oe-DCC. F, representative micrographs showing changes in mitochondrial Ca^2+^ in response to AgomiR-182/183 + oe-DCC measured by Ca^2+^ indicator Rhod-2 AM.
