# Supplementary figures and images for: Inhibition of MicroRNA-182/183 Cluster Ameliorates Schizophrenia by Activating the Axon Guidance Pathway and Upregulating DCC
Source: Oxid Med Cell Longev. 2022 Nov 10;2022:9411276. doi: 10.1155/2022/9411276 (PMC9671740; doi:10.1155/2022/9411276)

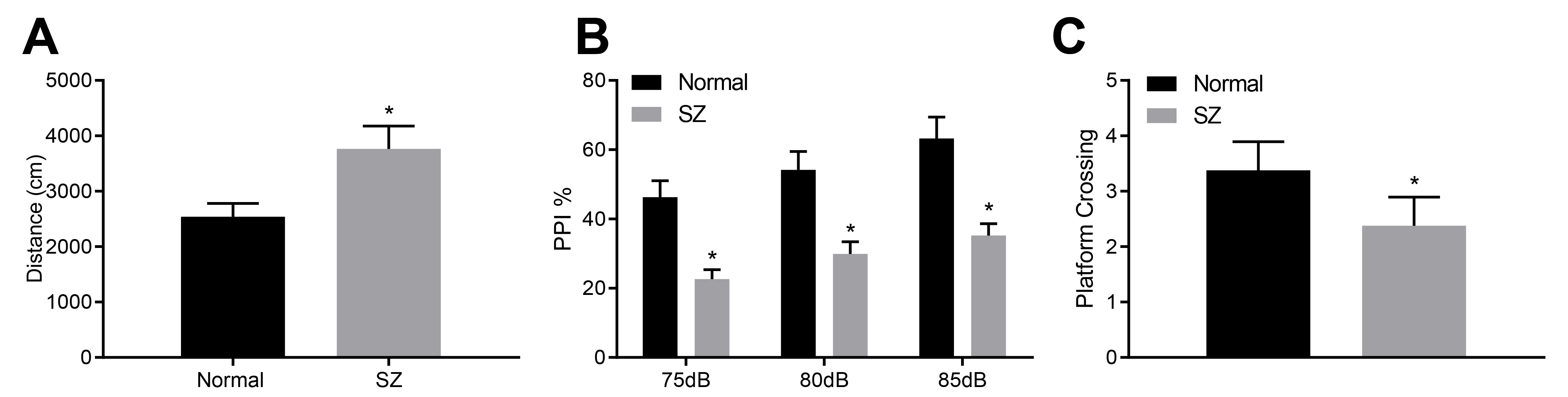

Supplement: Supplementary Materials — Supplementary Figure 1: characterization of SZ rat models by open field test. Supplementary Figure 2: representative image of stereotactic injection. Supplementary Figure 3: representative micrographs. Supplementary Table 1: relationship between miR-182/183 expression and clinicopathological characteristics of SZ patients. Supplementary Table 2: primer sequences for RT-qPCR. Supplementary Table 3: serum levels of NGF, BDNF, and GFAP in different treatment groups of SZ rats. [file 9411276.f1.zip › Fig S1.jpg]

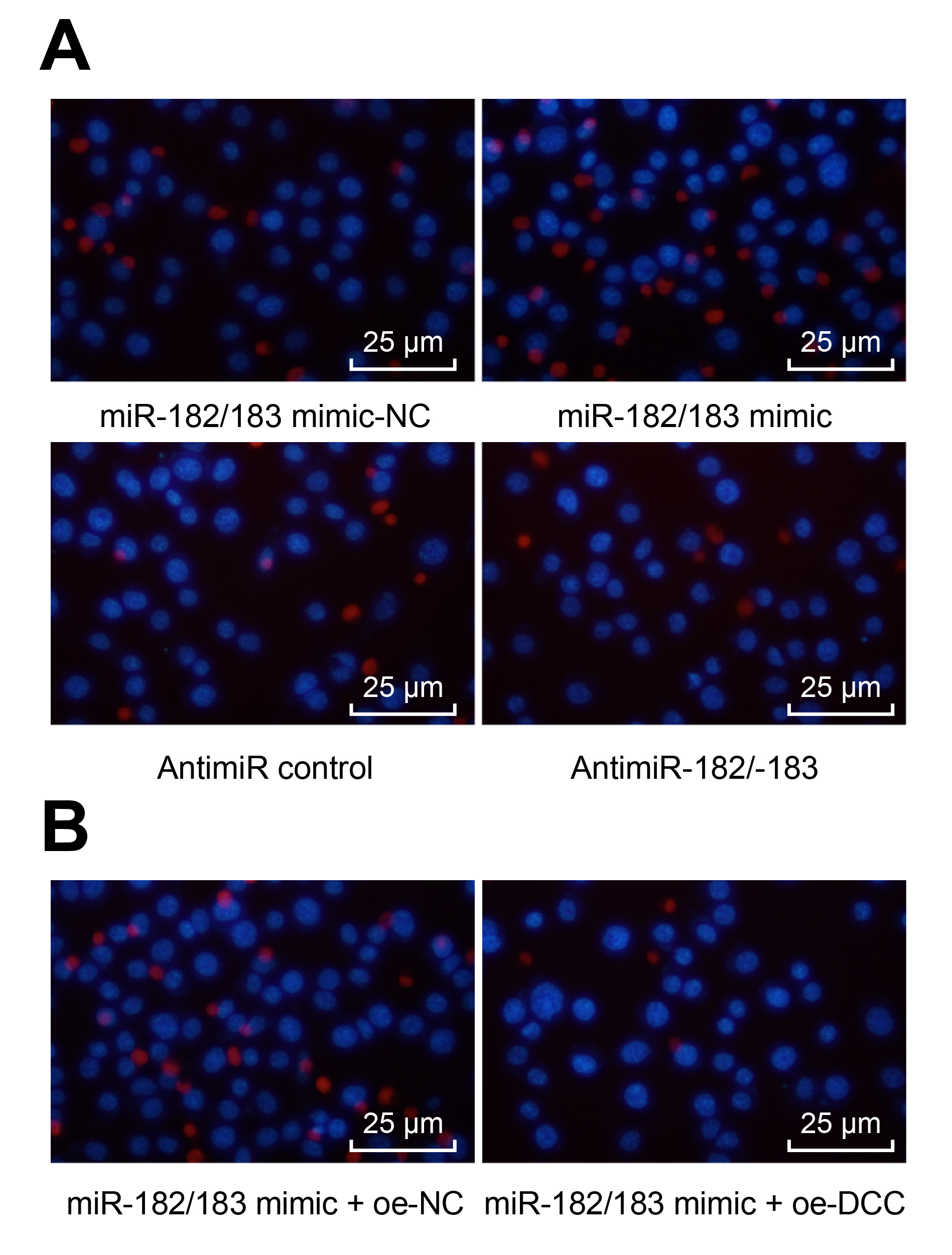

Supplement: Supplementary Materials — Supplementary Figure 1: characterization of SZ rat models by open field test. Supplementary Figure 2: representative image of stereotactic injection. Supplementary Figure 3: representative micrographs. Supplementary Table 1: relationship between miR-182/183 expression and clinicopathological characteristics of SZ patients. Supplementary Table 2: primer sequences for RT-qPCR. Supplementary Table 3: serum levels of NGF, BDNF, and GFAP in different treatment groups of SZ rats. [file 9411276.f1.zip › Fig S3.jpg]
